# PRMT5 acts as a tumor suppressor by inhibiting Wnt/β-catenin signaling in murine gastric tumorigenesis

**DOI:** 10.7150/ijbs.71581

**Published:** 2022-07-04

**Authors:** Yuling Tang, Lei Dong, Chong Zhang, Xiubin Li, Rongyu Li, Huisang Lin, Yini Qi, Mingchuan Tang, Yanli Peng, Chuan Liu, Jian Zhou, Ning Hou, Wenjia Liu, Guan Yang, Xiao Yang, Yan Teng

**Affiliations:** 1 State Key Laboratory of Proteomics, Beijing Proteome Research Centre, National Centre for Protein Sciences, Beijing Institute of Lifeomics, Beijing 102206, China.; 2 Laboratory Animal Center, the Academy of Military Medical Sciences, Beijing 100071, China.; 3 Department of Urology, the Third Medical Center of Chinese PLA General Hospital, Beijing 100039, China.

**Keywords:** PRMT5, gastric epithelium, tumorigenesis, Wnt/β-Catenin signaling

## Abstract

Previous studies have demonstrated the* in vitro* oncogenic role of protein arginine methyltransferase 5 (PRMT5) in gastric cancer cell lines. The *in vivo* function of PRMT5 in gastric tumorigenesis, however, is still unexplored. Here, we showed that *Prmt5* deletion in mouse gastric epithelium resulted in spontaneous tumorigenesis in gastric antrum. All *Prmt5*-deficient mice displayed intestinal-type gastric cancer within 4 months of age. Of note, 20% (2/10) of *Prmt5* mutants finally developed into invasive gastric cancer by 8 months of age. Gastric cancer caused by PRMT5 loss exhibited the increase in Lgr5^+^ stem cells, which are proposed to contribute to both the gastric tumorigenesis and progression in mouse models. Consistent with the notion that Lgr5 is the target of Wnt/β-catenin signaling, whose activation is the most predominant driver for gastric tumorigenesis, *Prmt5* mutant gastric cancer showed the activation of Wnt/β-Catenin signaling. Furthermore, in human gastric cancer samples, *PRMT5* deletion and downregulation were frequently observed and associated with the poor prognosis. We propose that as opposed to the tumor-promoting role of PRMT5 well-established in the progression of various cancer types, PRMT5 functions as a tumor suppressor *in vivo*, at least during gastric tumor formation.

## Introduction

Gastric cancer is the fifth most common type of cancer and the fourth leading cause of cancer mortality globally 1. Adenocarcinoma is the major type of gastric cancer and classified into two histologic subtypes: highly differentiated intestinal-type and poorly differentiated diffuse-type 2. The former occurs more frequently in the distal antrum and elderly patients (60%-80%) 3.

Gastric cancer is caused by the combination of genetic and environmental factors. Although many genes and related signaling pathways are altered in human gastric cancer, studies using genetically engineered mouse models verified that a few genetic mutants can initiate gastric tumor formation. Our previous study showed that 100% of mice with gastric epithelium specific loss of PTEN, which is frequently deleted in human gastric cancer, developed spontaneous gastric tumorigenesis as early as 2 months of age 4. When deleting both *PTEN* and *Smad4* in the gastric antral Lgr5^+^ stem cells, we observed the invasive intestinal-type gastric cancer in double mutant mice within as early as 3 months post induction 5. Another prominent driver for gastric carcinogenesis is the hyperactivation of Wnt/β-catenin signaling. Loss of function of adenomatous polyposis coli (APC), a negative regulator of β-catenin signaling, via either deletion or mutations, is sufficient to induce gastric tumorigenesis 6-8. Also, deletion of another Wnt negative regulator glycogen synthase kinase 3 (GSK3) or active mutation of β-catenin by deleting exon 3 in mouse gastric epithelium can efficiently lead to antral tumorigenesis 9. It is still under intense investigation that whether other genetic alternations are involved in gastric tumorigenesis.

Protein arginine methylation, a post-translational modification, is catalyzed by a class of enzymes called protein arginine methyltransferases (PRMTs) 10. PRMT5 is a prominent type II arginine methyltransferase that catalyzes symmetrical dimethylation of arginine residues in a variety of protein substrates 11, 12. It is widely-recognized that PRMT5 can promote the proliferation, invasion, and migration of various human cancer cells, including gastric cancers 13-16, breast cancers 17, colorectal cancers 18, and lung cancers 19. It is noteworthy that the evidence in support of the tumor-promoting function of PRMT5 were mainly inferred from* in vitro* studies 13-19. Therefore, it remained unexplored that what role PRMT5 plays in gastric cancer formation* in vivo*.

In the present study, we generated a gastric-specific *Prmt5* knockout mouse line. Unexpectedly, all* Prmt5* mutants developed spontaneous gastric tumor at antrum within 4 months of age, accompanied by the hyperactivation of Wnt/β-catenin signaling. Thus, we provided the first critical* in vivo* evidence for the causal relationship between PRMT5 downregulation and gastric tumorigenesis.

## Results

### Downregulation of PRMT5 in human gastric cancer

**Figure 1:**
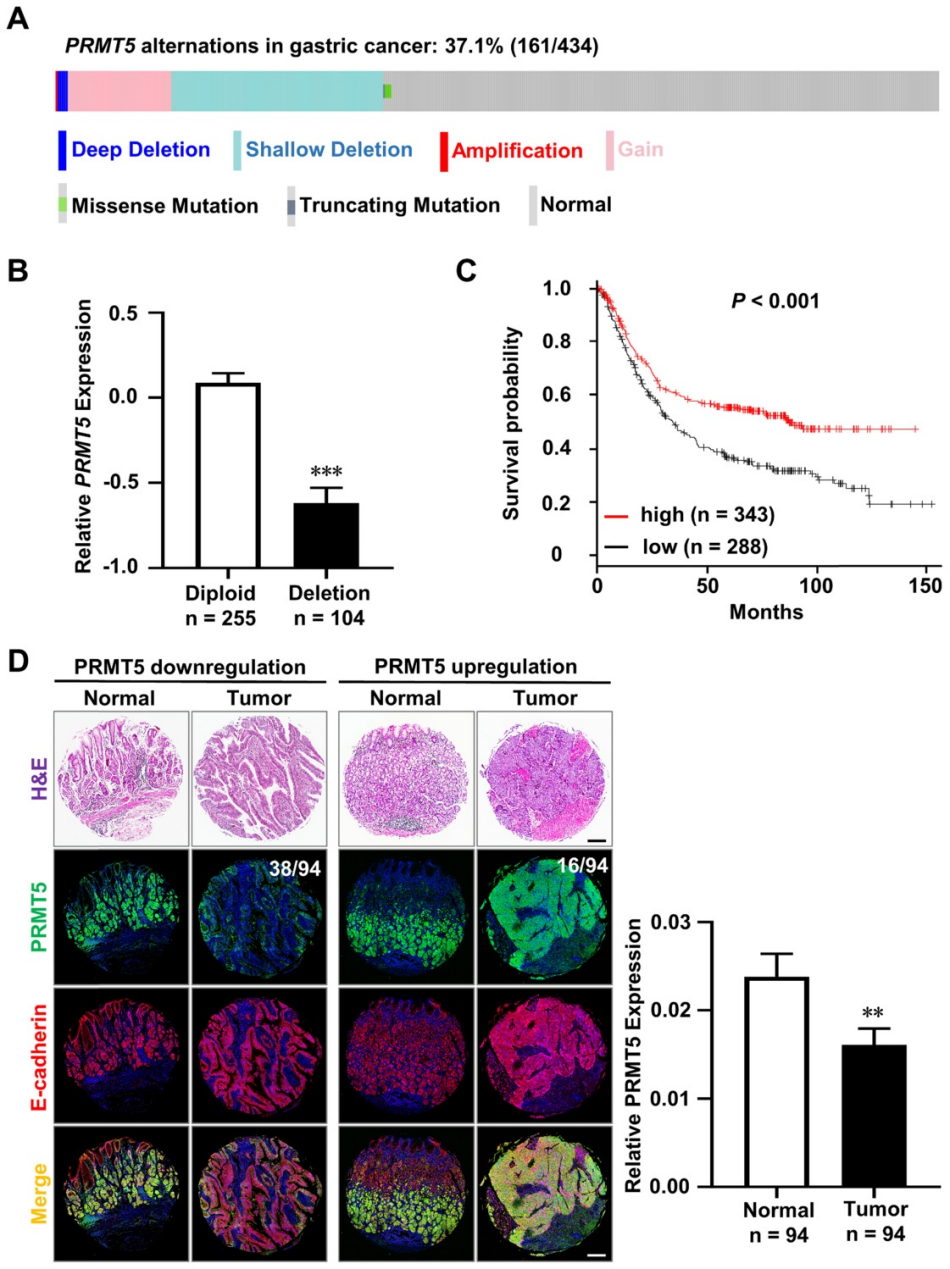
** Downregulation of PRMT5 in human gastric cancers. A.** The total alterations of the *PRMT5* gene, including deletions, gain-of-functions, amplifications, and mutations, were revealed in 37.1% (161/434) of human primary gastric cancer samples by analyzing TCGA database (deep deletion, n = 5; shallow deletion, n = 104; gain, n = 51; amplification, n = 1; mutations, n = 3). **B.** Deletion of *PRMT5* gene in human gastric cancer samples was tightly associated with its decrease in mRNA level. Data were represented as means ± SEM (Diploid, n = 255; Deletion, n = 104. *** *P* < 0.001, Student's t-test). **C.** Kaplan-Meier curves revealed that the decreased PRMT5 expression was linked to a poor outcome in human gastric cancer patients (n = 631). Parameters: Affy id/Gene symbol, PRMT5 1564520_s_at. Split patients by median; survival, OS; follow-up threshold, all; Lauren classification, all. *** *P* < 0.001. Significance was calculated using log-rank test. **D.** H&E staining and Co-immunofluorescence staining (Co-IF) for PRMT5 (green) and E-cadherin (red) in the human tissue microarray representing 94 patients with intestinal-type gastric cancer and adjacent normal tissues. PRMT5 expression was downregulated in 40.4% (38/94) of gastric cancer samples compared to those of adjacent normal tissues, while PRMT5 expression was upregulated in 17% (16/94) of gastric cancer samples. Low PRMT5 expression was associated with intestinal-type gastric cancer. Data were represented as means ± SEM (n = 94 pairs, ** *P* < 0.01, one-way ANOVA test). Scale bar, 200 µm.

We analyzed the alterations of *PRMT5* gene in 434 human primary gastric cancer samples from TCGA database 20-23. We found that* PRMT5* deletion, including shallow deletion and deep deletion, occurred in 25.1% (109/434) of human gastric cancer samples, which was more prevalent than gain 11.8% (51/434) and amplification 0.2% (1/434), indicating that PRMT5 loss might be implicated in gastric cancer formation and progression (Figure 1A). As anticipated, *PRMT5* deletion was tightly associated with its decrease in mRNA level (Figure 1B, diploid, n = 255; deletion, n = 104, *P* < 0.001). Furthermore, we discovered that the decreased PRMT5 expression was linked to a poor outcome for gastric cancer (Figure 1C, n = 631, *P* < 0.001). This indicated that PRMT5 may act as a tumor suppressor in gastric cancer.

In parallel, we examined PRMT5 protein level in a human tissue microarray representing 94 patients with intestinal-type gastric cancer and adjacent normal tissues. To better exhibit the gastric epithelium, we performed co-immunofluorescence staining (Co-IF) for PRMT5 (green) and E-cadherin (red). The protein level of PRMT5 was calculated by the mean optical density values of immunofluorescence. The ratio of tumor versus adjacent normal tissue > 2 was defined by “upregulation” and the ratio < 0.5 was defined by “downregulation”. We found that 40.4% (38/94) of paired samples showed a lower expression of PRMT5 in the gastric cancer compared to the adjacent normal tissues (Figure 1D). We also found that PRMT5 upregulation in 17% (16/94) gastric cancer samples (Figure 1D). Statistical analysis showed that low PRMT5 expression is associated with gastric cancer (Figure 1D, n = 94, *P* < 0.01).

### *Prmt5* deletion gave rise to invasive intestinal-type gastric cancer in antrum

**Figure 2:**
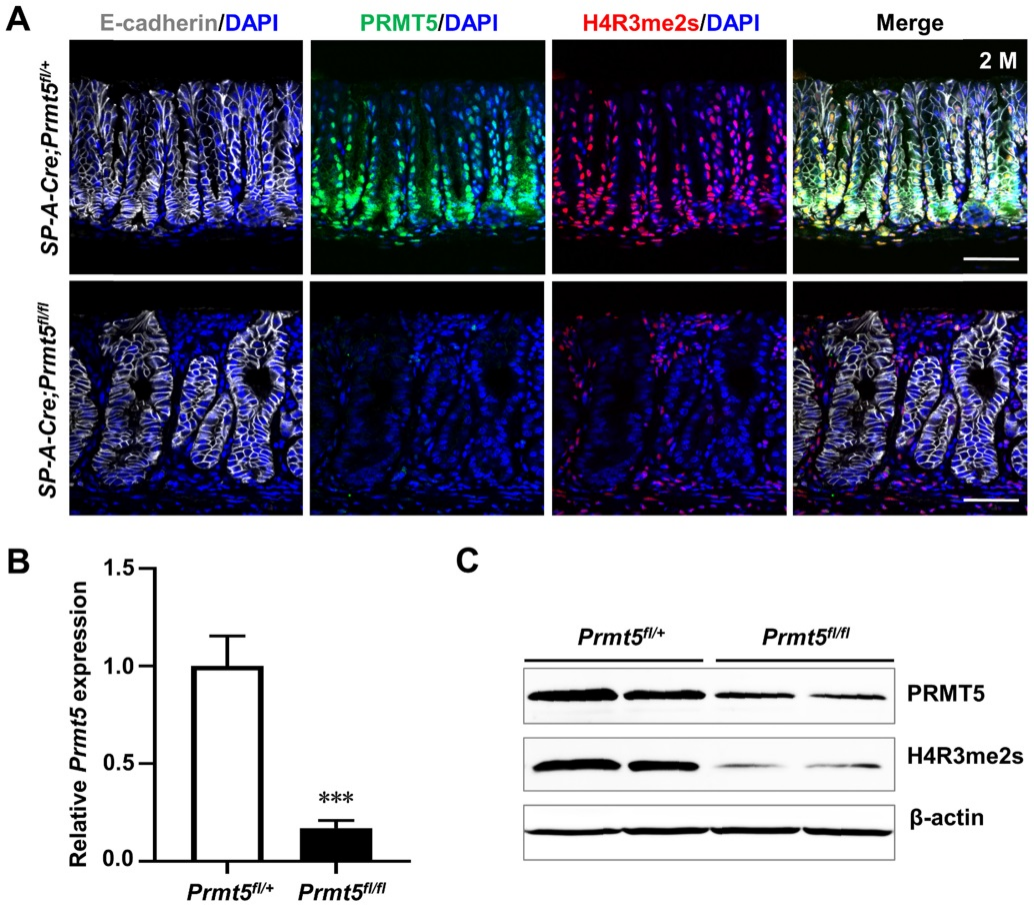
***Prmt5* was efficiently deleted in the gastric antral epithelium. A.** Co-immunofluorescence staining (Co-IF) for E-cadherin (white), PRMT5 (green), and H4R3me2s (red) in the antrum of *SP-A-Cre;Prmt5^fl/+^* (hereafter control) and *SP-A-Cre;Prmt5^fl/fl^* mice (hereafter mutant) at 2 months of age. E-cadherin labeled the epithelium. Scale bar, 50 µm. **B.** RT-qPCR analysis of *Prmt5* mRNA level in control and *Prmt5* mutant antrum at 2 months of age. Data were represented as means ± SEM (n = 4, *** *P* < 0.001, Student's t-test). **C.** Western blot analysis of PRMT5 and H4R3me2s expression in the extract of antrum from control and mutant mice at 2 months of age. β-actin was used as loading controls. n = 4.

To investigate the role of PRMT5 in gastric tumorigenesis* in vivo*, we generated gastric-specific *Prmt5* knockout mice. Strategically, *Prmt5*-conditional knockout mice (*Prmt5^flox/flox^* mice) were crossed with* SP-A-Cre* transgenic mice, where Cre-mediated recombination begins from embryonic day 16.5 (E16.5) 24. We first examined the deletion of *Prmt5* in *SP-A-Cre;Prmt5^fl/+^* (here after control) and *SP-A-Cre;Prmt5^fl/fl^* (here after mutant) mice. In control mice, PRMT5 was mainly restricted to the lower region of antral glands (Figure 2A), where the actively proliferating cells reside 25, 26. In the *Prmt5* mutant antral epithelium, PRMT5 expression was significantly reduced both at the mRNA and protein levels, as determined by real-time quantitative PCR (RT-qPCR), Western blot, and immunohistochemical (IHC) analyses (Figure 2A-C). As expected, we found the significantly downregulation of a repressive histone mark H4R3me2s (Figure 2A, 2C), which is well-known to be catalyzed by PRMT5 27-30.

**Figure 3:**
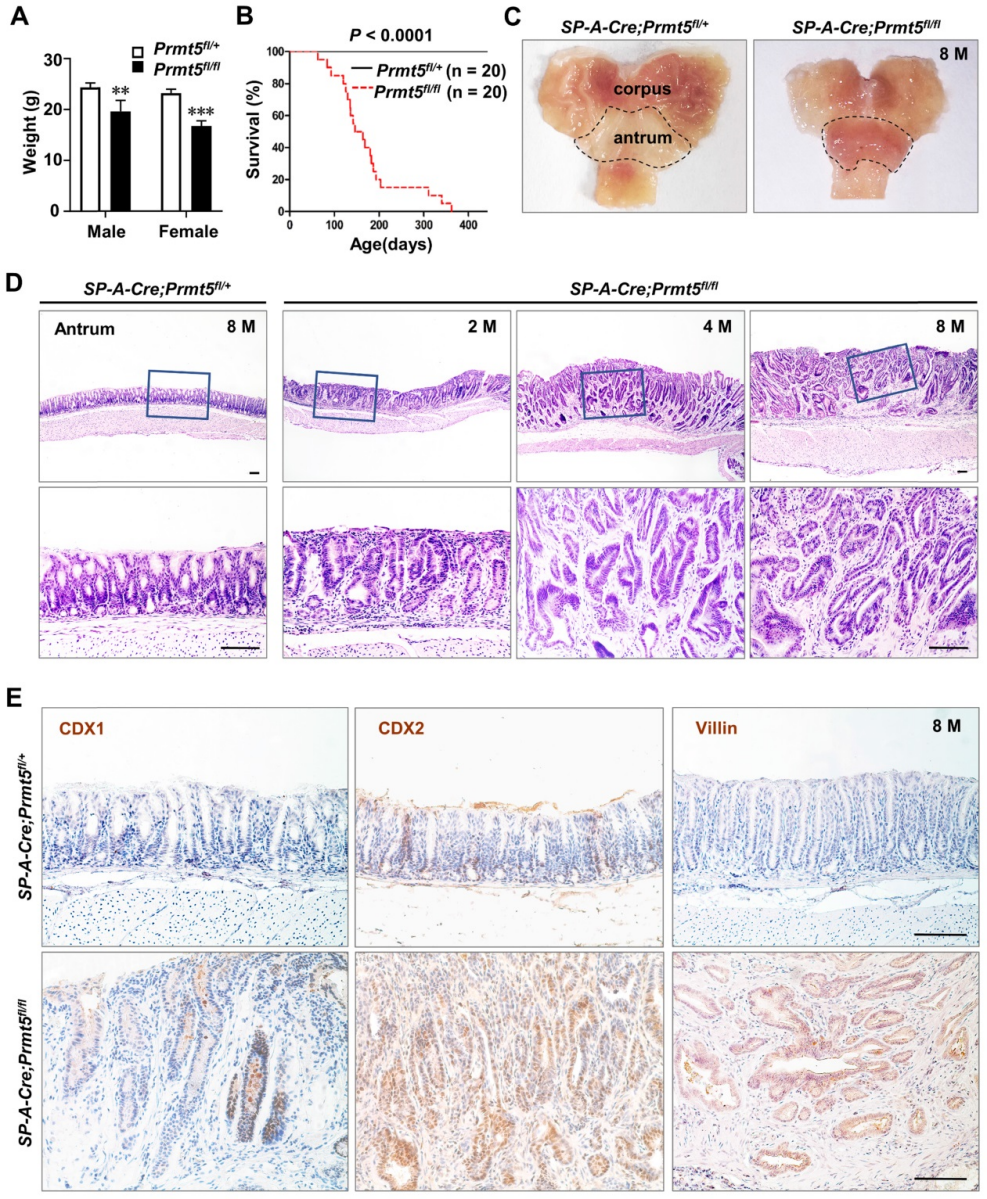
***Prmt5* deletion resulted in gastric tumorigenesis in mice. A.** *Prmt5* deletion resulted in significant weight loss. Body weight were represented as means ± SEM (n = 4, ** *P* < 0.01, *** *P* < 0.001, Student's t-test). **B.** *Prmt5* deletion resulted in a reduced survival. Kaplan-Meier survival curve of *Prmt5* mutant mice (red dotted line; n = 20) compared with control mice (black solid line; n =20. *** *P* < 0.001, log-rank test). **C.** *Prmt5* mutant mice exhibited markedly thickened antral epithelium. Gross anatomy of the representative stomach from 8-month-old control and mutant mice. The dotted line indicated the antral region. **D.** Representative H&E staining of antral epithelium from 8-month-old (n = 10) control along with 2-month-old (n = 5), 4-month-old (n = 13), and 8-month-old (n = 10) *Prmt5* mutant mice. The blue solid-line boxes were magnified underneath. Scale bar, 100 µm. **E.** IHC of CDX1, CDX2 and Villin in 8-month-old control and mutant antrum. Scale bar, 100 µm.

All* SP-A-Cre;Prmt5^fl/fl^* mice were born normally. At two months of age, however, all *Prmt5* mutant mice became weaker and resulted in significant weight loss (Figure 3A). Furthermore, the mutant mice began to die at the age of 2 months and none of them survived more than a year, while all control mice were alive (Figure 3B). Macroscopically, *Prmt5* mutant mice exhibited markedly thickened antral epithelium (Figure 3C, dotted line). Histologic examination showed clearly defined gastric units in the antrum of control mice at any age (Figure 3D). By contrast, all *Prmt5* mutant mice exhibited multistage process of the antral tumorigenesis, with hyperplasia at 2 months, microadenoma at 4 months, and gastric cancer at the age of 8 months (Figure 3D). Furthermore, gland structure in antral tumors (Figure 3D), together with the ectopic expression of CDX1, CDX2 and Villin, the well-known intestinal epithelial markers (Figure 3E), suggested that gastric cancer caused by *Prmt5* deletion was intestinal-type gastric cancer.

**Figure 4:**
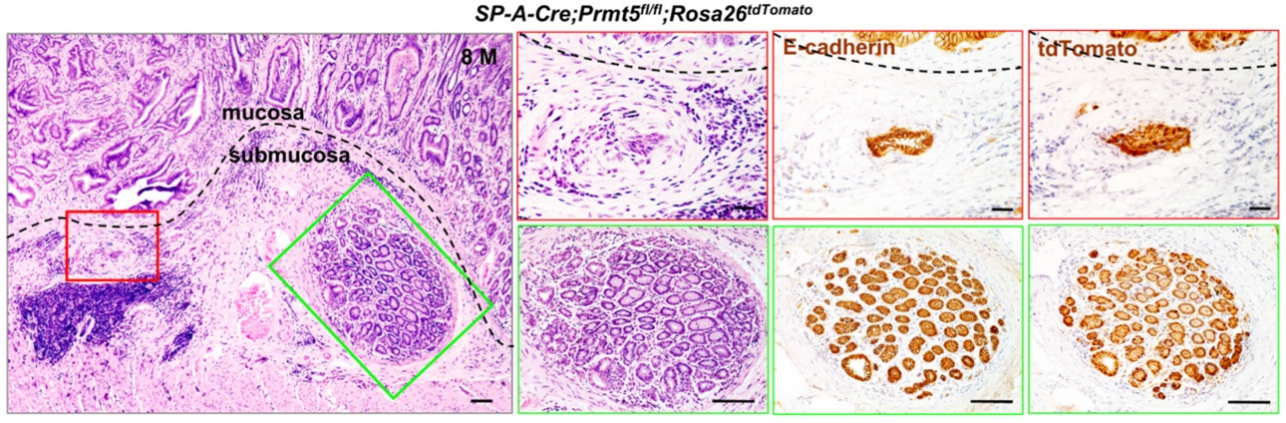
** Loss of PRMT5 resulted in invasive gastric cancer.** H&E staining and IHC of E-cadherin and tdTomato in the invasive gastric cancer of* SP-A-Cre;Prmt5^fl/fl^;Rosa26^tdTomato^* mice at 8 months of age. A black dotted line demarcated two regions for the mucosa and submucosa. The red and green solid-line boxes were magnified on the right, respectively. Scale bar, 100 µm.

In particular, 20% (2/10) of *Prmt5* mutant mice developed invasive gastric cancer that invaded into submucosa layer, as evidenced by H&E and the expression of E-cadherin (Figure 4). To examine whether the invading gastric epithelial cells resulted from *Prmt5* deletion, *SP-A-Cre;Prmt5^fl/+^* mice were bred with a Cre reporter mouse line *Rosa26-LoxP-Stop-LoxP-tdTomato (Rosa26^tdTomato^)* for two generations, which could permanently label the recombinant cells and their progeny with the red fluorescent protein variant tdTomato 31. As expected, the invasive cells in submucosa layer were tdTomato-positive (Figure 4), demonstrating that *Prmt5* mutant cells contributed to the occurrence and the progression of gastric tumors. Taken together, these results revealed a causal relationship between the loss of PRMT5 and antral tumorigenesis, supporting a tumor-suppressor role of PRMT5 *in vivo*, at least in antrum. Since SP-A-Cre is also expressed in lung, we performed H&E staining of lung tissue in *SP-A-Cre;Prmt5^fl/+^* and *SP-A-Cre;Prmt5^fl/fl^* mice at 10 months of age. No morphological abnormity was found in lung of *Prmt5* mutant mice (Figure S1).

### *Prmt5* deletion led to the increased proliferation of antral epithelium

**Figure 5:**
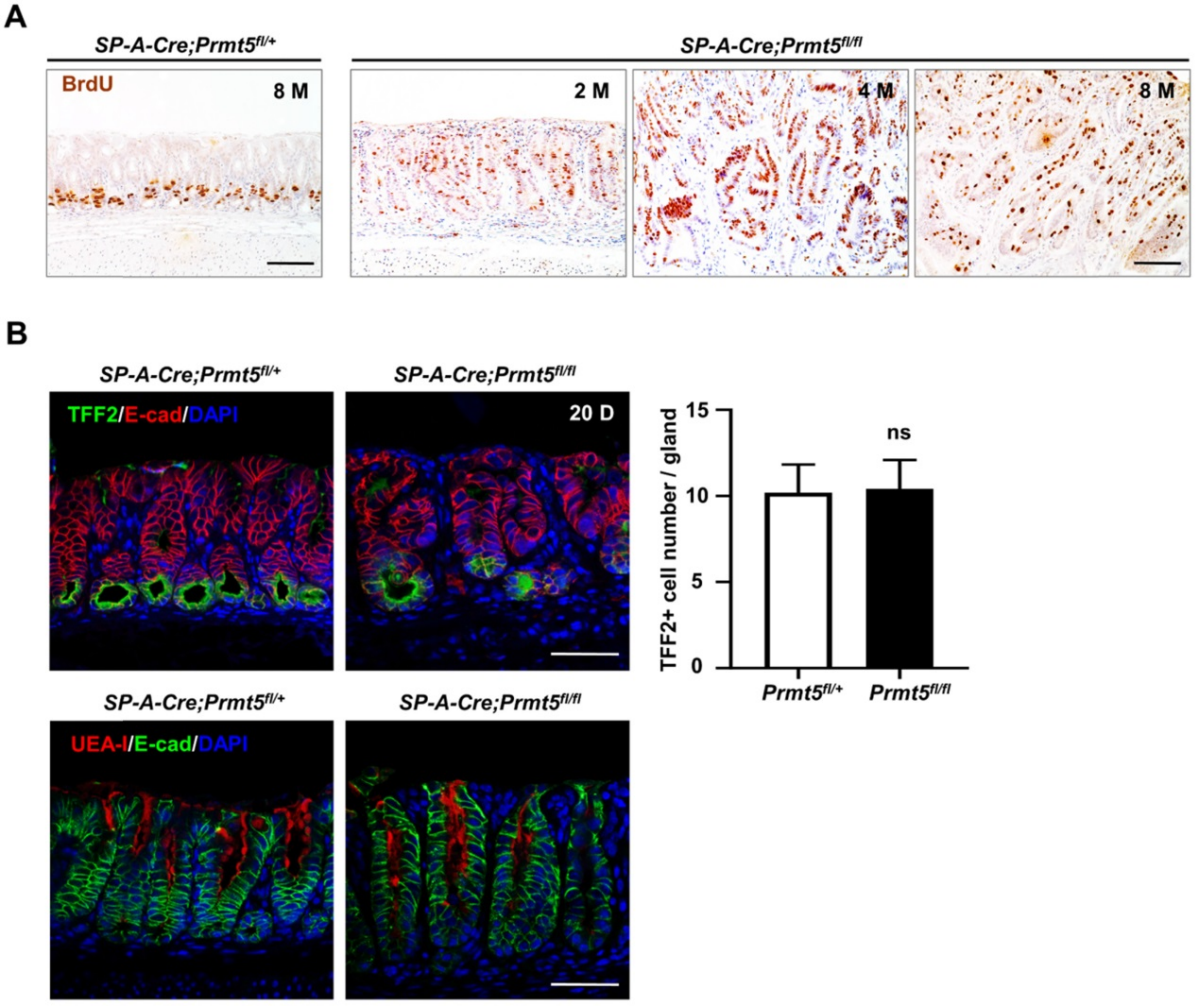
***Prmt5* deletion increased cell proliferation without affecting the differentiation of antral epithelium. A.** Representative BrdU IHC of antrum in 8-month-old control and 2-month-old, 4-month-old, and 8-month-old *Prmt5* mutant mice. Scale bars, 100 µm. **B.** Representative IF analysis of two differentiated cell markers ulex europaeus agglutinin type 1 lectin (UEA-I, for surface mucous cells) and trefoil family factor 2 (TFF2, for mucous neck cells) in 20-day-old control and mutant mice. Scale bar, 50 µm. Quantification of TFF2^+^ cell number per gland was represented as means ± SEM; Student's t-test; ns, not significant.

Gastric tumorigenesis at the cellular level results from the disorders of cell proliferation or differentiation, or both. 100% of *Prmt5* mutant mice developed hyperplasia as early as 2 months of age (Figure 3D). Hyperplasia as an initial step of tumorigenesis is closely related to enhanced cellular proliferation. We performed BrdU incorporation assay in control and mutant mice. In the control antrum, BrdU-positive cells were confined to the base of the gastric glands (Figure 5A), where antral stem cells such as Lgr5^+^ cells and their progeny transient proliferating cells localize 25. By contrast, in mutant antrum, BrdU-positive cells significantly increased and expanded extensively throughout the whole mucosa as early as 2 months of age (Figure 5A).

We next investigated whether *Prmt5* deletion affect maturation or differentiation of antral epithelium. At postnatal 20 days (P20), immunohistochemical staining showed that the expression of both surface mucous cells marker ulex europaeus agglutinin type 1 lectin (UEA-I) and mucous neck cells marker trefoil family factor 2 (TFF2) were comparable between control and mutant antral glands (Figure 5B). Overall, gastric tumorigenesis caused by PRMT5 loss was mainly attributable to the increase in cell proliferation, especially in actively proliferating gland epithelium.

### Lgr5^+^ stem cells were dramatically increased in the invasive gastric cancer caused by PRMT5 loss

**Figure 6:**
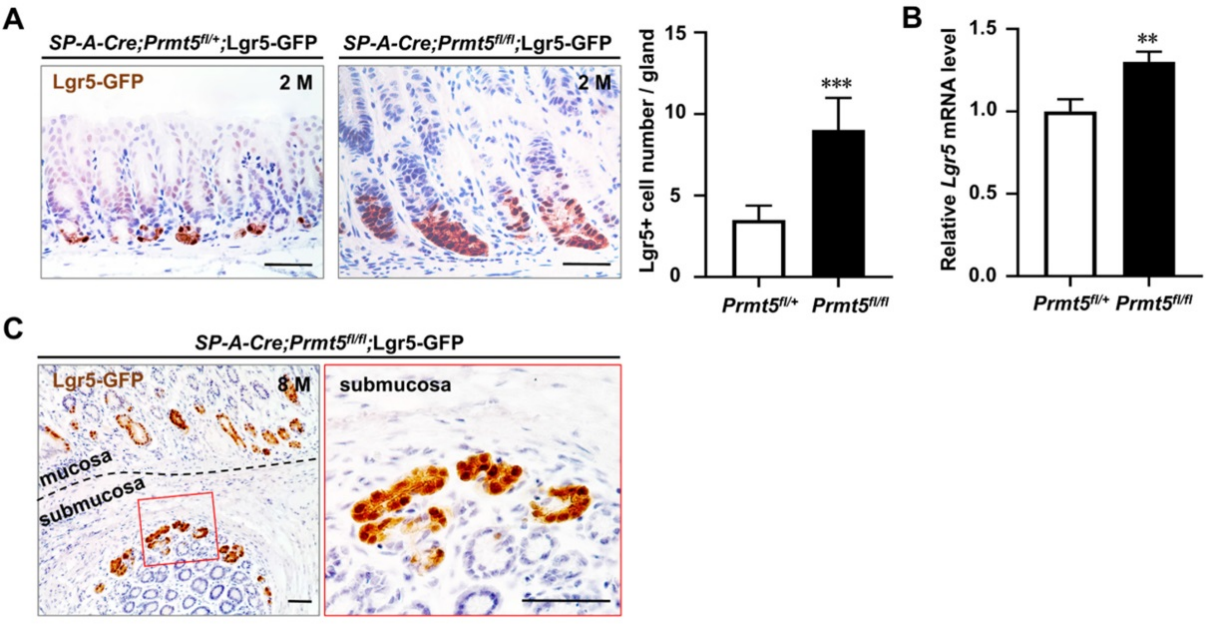
** PRMT5-loss-induced invasive gastric cancer showed an increase in the number of Lgr5^+^ cells in mucosa and submucosal regions. A.** GFP-positive Lgr5^+^ cells were significantly increased in *Prmt5* mutant epithelium compared to those of control mice at 2 months of age. Representative IHC for Lgr5-GFP expression in control and mutant mice at 2 months of age. Quantification of the Lgr5^+^ cell number per gland was presented as means ± SEM (n = 4, ***P < 0.001, Student's t-test). Scale bar, 50 µm. **B.** Endogenous* Lgr5* mRNA level was upregulated in *Prmt5* mutant mice. The relative level of *Lgr5* were measured using RT-qPCR in the control and mutant mice at 2 months of age. Data were represented as means ± SEM (n = 4, ** *P* < 0.01, Student's t-test). **C.** GFP-expressing Lgr5^+^ cells were observed in the invasive regions of gastric cancer. Representative IHC for Lgr5-GFP expression in the invasive regions of gastric cancer of *Prmt5* mutant mice at 8 months of age. A black dotted line demarcated two regions for the mucosa and submucosa. The red solid-line box was magnified on the right. Scale bar, 50 µm.

The marked increase in proliferation of antrum after *Prmt5* knockout prompted us to investigate the potential alteration of stem/progenitor cells. Lgr5, an orphan G protein coupled receptor, has been identified as a marker of frequently cycling gastric epithelial stem cells residing at the base of the antral gland 25, 32. Our and other groups have demonstrated that mutant Lgr5^+^ cells can initiate gastric cancer and accelerate the progression and metastasis of gastric cancer 5, 33-35. We detected the Lgr5^+^ cell number in *Prmt5* mutant mice. Due to the lack of Lgr5 antibody, Lgr5^+^ cells *in situ* were visualized with enhanced green fluorescent protein (GFP) through further breeding with* Lgr5-eGFP-IRES-CreERT2* knock-in mice 25. We found that GFP-positive Lgr5^+^ cells were significantly increased in *Prmt5* mutant epithelium compared to that of control mice at 2 months of age (Figure 6A). Consistently, RT-qPCR results showed an increase in *Lgr5* mRNA level in the antrum of mutant mice at 2 months of age (Figure 6B). Of note, GFP-expressing Lgr5^+^ cells were also observed in the invasive regions of gastric cancer (Figure 6C). To sum up, at the cellular level, deletion of *Prmt5* increased the number of Lgr5^+^ cells both in mucosa and submucosal area, which may further promote the development and progression of gastric cancer.

### *Prmt5* deletion activated Wnt/β-catenin signaling

Besides being a marker for gastric stem cells, Lgr5 is also a well-known gastrointestinal target of Wnt/β-catenin signaling 36, whose activation is a critical cause of gastric tumorigenesis. We next investigated whether Wnt/β-catenin signaling was hyperactivated in *Prmt5* deletion-induced gastric cancer.

**Figure 7:**
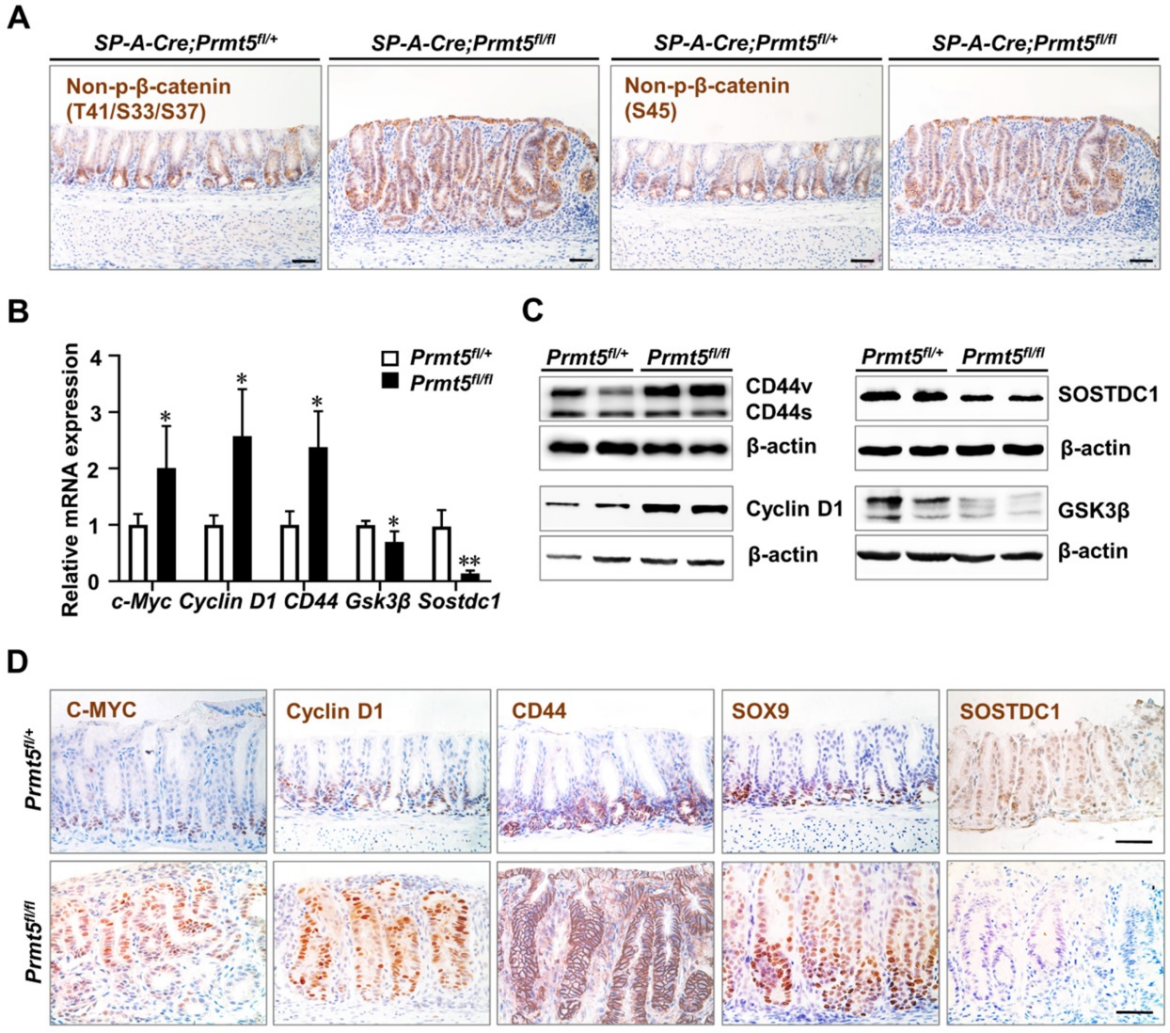
***Prmt5* deletion activated Wnt/β-catenin signaling. A.** IHC analysis of the control and mutant antrum at 2 months of age with antibodies for Non-phospho-β-catenin^Ser45^ and Non-phospho-β-catenin^Ser33/37/Thr41^. Scale bar, 50 µm. **B.** The relative level of *c-Myc, Cyclin D1, CD44, Gsk3β*, and* Sostdc1* were measured using RT-qPCR in the control and mutant mice at 2 months of age. Data were represented as means ± SEM (n = 4, * *P* < 0.05, ** *P* < 0.01, Student's t-test). **C.** The protein level of CD44, Cyclin D1, GSK3β, and SOSTDC1 were measured using Western blot analysis in the control and mutant mice at 2 months of age (n = 3). The two bands of CD44 indicated the variant (CD44v) and standard (CD44s) forms respectively. **D.** IHC analysis of the control and mutant antrum at 2 months of age with antibodies for C-MYC, Cyclin D1, CD44, SOX9, and SOSTDC1. Scale bar, 50 µm.

β-catenin protein is a core component of the Wnt signaling pathway 37. We examined the protein level of the non-phosphorylated β-catenin, which indicates the activation of Wnt signaling pathway 38. We found that non-phosphorylated β-catenin at Ser45 and Ser33/37/Thr41 sites were significantly upregulated in *Prmt5* mutant epithelium (Figure 7A).

We further detected the gene expression of some Wnt/β-catenin signaling targets including C-MYC, Cyclin D1, and CD44, all of which are associated with human gastrointestinal cancers 39-41, in the control and mutant mice at the age of 2 months. C-MYC and Cyclin D1 are upregulated in human gastric cancer and linked with malignant progress and poor survival 42, 43. And *c-Myc* transgenic mice can develop gastric tumorigenesis within 25 weeks 44, 45. We found that the mRNA levels of *c-Myc* and *Cyclin D1* were much higher in *Prmt5* mutant epithelium than controls (Figure 7B), and Western blot analysis showed that *Prmt5* deletion increased CyclinD1 protein expression (Figure 7C). IHC staining showed that as opposed to the confined localization of C-MYC and Cyclin D1 at the base of glands in control mice, their expression expanded upward throughout the whole epithelium in *Prmt5* mutant mice (Figure 7D). CD44 is often upregulated in gastric cancer and associated with increased metastatic potential and poor survival 46. CD44 is also a candidate marker for gastric cancer stem cells 47, and more importantly, deletion of *CD44* suppressed gastric cancer progression in a transgenic mouse model 48. We found that the expression of *CD44* was elevated in the* Prmt5* mutant antrum compared to the control antrum by the RT-qPCR assays (Figure 7B). IHC result revealed that CD44-positive cells occupied the bottom of control glands, whereas in mutant mice, CD44-positive cells expanded to the upper region of the glands (Figure 7D). Of note, in the Western blot result of CD44, we found that CD44 variant (CD44v) but not CD44 standard (CD44s) was significantly increased in *Prmt5* mutant mice (Figure 7C). Previous studies have shown that CD44v is the dominant form of CD44 in the human gastric cancer tissue, and more importantly, CD44v but not CD44s increased the frequency of tumor initiation in immunocompromised mice 48-50. Therefore, the significant increase in CD44v may contribute to the gastric tumorigenesis in *Prmt5* mutant mice. SOX9 is not only a downstream target of Wnt/β-catenin signaling, but also a positive regulator of Wnt/β-catenin signaling 51, 52. SOX9 expression is elevated in human gastric cancer samples and correlated with poor clinical outcomes 53-55. In several human gastric cancer cell lines, SOX9 knockdown suppressed tumor growth by inhibiting Wnt/β-catenin signaling 54. IHC showed that SOX9-expressing cells widely spread in *Prmt5* mutant mice but restricted to the lower part of the control antral glands (Figure 7D).

Next, we detected the expression of glycogen synthase kinase 3β (GSK3β) and sclerostin domain containing 1 (SOSTDC1), two negative regulators of Wnt/β-catenin signaling 56. GSK3β protein levels are decreased in human gastric cancer samples 56, 57. Of note,* GSK3* deletion alone in mouse antral epithelium is sufficient to induce tumor formation through activating Wnt/β-catenin signaling 9. We found that *Prmt5* mutant mice exhibited a decrease in GSK3β expression, as shown by RT-qPCR and Western blot analyses (Figure 7B-C). SOSTDC1 is frequently depleted in gastric cancers and associated with poor outcomes 58, 59. SOSTDC1 plays a tumor-suppressive role in gastric cancer cells, as SOSTDC1 knockdown accelerates tumor growth and metastasis 59. As expected, SOSTDC1 expression was significantly downregulated in *Prmt5* mutants compared with control mice (Figure 7B-D).

Taken together, *Prmt5* deletion-induced gastric cancer exhibited the hyperactivation of Wnt/β-catenin signaling, recapitulating the molecular alterations in human gastric cancer.

## Discussion

Herein, we provide the first critical* in vivo* role of PRMT5 in suppressing gastric cancer formation. This seems to be contrary to the notion that PRMT5 can promote tumor growth and progression in a variety of human cancer cells including gastric cancers 11, 13-19. Of note, the tumor-promoting function of PRMT5 was observed in the *in vitro* experiments of cancer cell lines 13-19. These cancer cell lines are isolated from human tumor samples, so they mainly reflect the nature of the cancer progression stage rather than the formation stage. In fact, we also found that knockdown of PRMT5 in some gastric cancer lines with high level of PRMT5 did inhibit tumor growth. Therefore, like TGF-β signaling 60, PRMT5 may exert the opposite roles in the tumorigenesis and progression at least in gastric cancer. Given that PRMT5 as a major type II arginine methyltransferase has diverse substrates in different context 61, PRMT5 may regulate distinct substrates in different stages. Another possibility is the heterogeneity of human gastric cancer. *PRMT5* gene deletion was found in approximately 25% of human gastric cancer samples. We propose that in these gastric cancer samples, low level of PRMT5 caused by gene deletion leads to or contributes to gastric tumorigenesis. However, it cannot be ruled out the possibility that PRMT5 overexpression can also lead to gastric tumorigenesis although it is needed to investigate using *in vivo* mouse models. Nevertheless, since a few PRMT5 inhibitors are currently being tested in clinical trials (e.g., GSK3326595 and JNJ-64619178) 62, 63, our findings raised a concern about PRMT5 being a therapeutic target of cancer, at least in gastric tissue. In addition, it is needed to examine in the future whether PRMT5 functions as a tumor suppressor in other tumor-type formations using *Prmt5* knockout mouse models.

In the present study, we found that the hyperactivation of Wnt/β-catenin signaling in *Prmt5* mutant antral epithelium as evidenced by several lines: the alterations in RNA level of *c-Myc, Cyclin D1, CD44, Gsk3β, Lgr5*, and *Sostdc1*, all of which led to the corresponding protein changes; the increase in the protein level of SOX9; the upregulation of non-phosphorylated β-catenin at Ser45 and Ser33/37/Thr41 sites. Therefore, we demonstrate that PRMT5 could inhibit the hyperactivation of Wnt/β-catenin signaling in normal antral epithelium. Previous *in vitro* studies from various cancer cell lines, however, suggest that PRMT5 could promote the activation of Wnt/β-catenin signaling 17, 64-69. Again, given the opposite roles of PRMT5 in the tumorigenesis and progression at least in gastric cancer, PRMT5 may regulate different downstream effectors to inhibit or activate Wnt/β-catenin signaling in different context. Indeed, a previous study showed that in murine normal cardiomyocytes, PRMT5 could inhibit the Wnt/β-catenin pathway to protect against pathological cardiac hypertrophy 70. Taken together with our findings, these two results indicated that PRMT5 could inhibit the Wnt/β-catenin pathway under normal circumstance at least in gastric epithelium and cardiomyocytes. Interestingly, we also found that PRMT5 was highly expressed in the lower part of antral glands, where Lgr5^+^ stem cells and its direct progeny transient proliferating cells localize 25, 71. One feature of these cells was actively proliferating, consistent with the moderate activation of Wnt/β-catenin signaling in these cells.* Prmt5* deletion led to the extensive expansion of proliferating cells upward, which was accompanied with hyperactivation of Wnt/β-catenin signaling. Therefore, we assumed that PRMT5 might be a brake of Wnt/β-catenin signaling in antral epithelium. In the future, it is needed to investigate how PRMT5 inhibits Wnt/β-catenin signaling in gastric epithelium. A clue came from the cellular localization of PRMT5. We found that PRMT5 was localized to both the nuclei and cytoplasm of antral epithelium. Given that the roles of cytoplasmic and nuclear PRMT5 are pleiotropic, it is proposed that, in normal antral epithelium, PRMT5 can inhibit the hyperactivation of Wnt/β-catenin signaling through multiple mechanisms directly or indirectly.

## Materials and Methods

### Mice

*Prmt5^fl/fl^* mice,* SP-A-cre* mice were described earlier 72, 73. The Jackson Laboratory provided the *Lgr5-eGFP-IRES-Cre^ERT2^* and *Rosa26-loxP-stop-loxP-tdTomato* mice 36, 74. Mice were injected intraperitoneally with 100 μg BrdU (B5002, Sigma Aldrich) per gram of body weight 2 hours before harvesting for BrdU-labeling experiments. All animals were maintained under specific pathogen-free conditions, and animal experiments were approved by the Animal Experiment Committee of the Institute of Biotechnology.

### Histology and immunohistochemistry assay

Mouse gastric tissues were fixed in 4% paraformaldehyde (PFA) overnight and then embedded in paraffin. Five-micrometer-thick slices were cut from paraffin blocks and placed on coated slides for the following hematoxylin and eosin (H&E), immunohistochemistry (IHC) and immunofluorescence (IF) staining. As for the tissue microarray of human gastric cancer, it was purchased from Xi'an Best Biotechnology Ltd., Co. (ST2084a, https://www.alenabio.com).

After deparaffinization and rehydration, H&E staining was performed using standard techniques. As for IHC and IF staining, 3% H_2_O_2_ was needed for neutralizing endogenous peroxidase activity. Heat treatment with citrate solution (pH 6.0) was used to unmask the antigen, followed by blocking with goat serum for 1 hour at 37°C to decrease nonspecific antibody binding. Immunohistochemical staining for BrdU requires additional diluted hydrochloric acid solution treatment for 20 minutes. Then the slices were incubated overnight at 4°C with the following primary antibodies: PRMT5 (1:100, Abcam, ab109451), H4R3me2s (1:500, Abcam, ab5823), E-cadherin (1:300, BD Biosciences, 610181), CDX1 (1:200, Sigma Aldrich, HPA055196), CDX2 (1:200, Sigma Aldrich, SAB4301787), Villin (1:200, Santa Cruz, sc-7672), RFP (1:500, Rockland, 600-401-379; the RFP antibody can recognize tdTomato), BrdU (1:300, Abcam, ab6326), TRITC-labelled UEA-I (1:100, Sigma, L4889), TFF2 (1:500, Abcam, ab203237), GFP (1:200, Cell Signaling Technology, 2956), Non-phospho-β-catenin^Ser45^ (1:200, Cell Signaling Technology, 19807), Non-phospho-β-catenin^Ser33/37/Thr41^ (1:200, Cell Signaling Technology, 8814), c-Myc (1:100, Abcam, ab32072), Cyclin D1 (1:500, Abcam, ab134175), CD44 (1:4000, Abcam, ab189524), SOX9 (1:2000, Abcam, ab185230), SOSTDC1 (1:100, Abcam, ab99340). Then we added the secondary antibodies (Beijing Zhongshan Golden Bridge Biotechnology Co.) and incubated at room temperature for 1 hour. IHC was observed by DAB (Zhongshan Biotech, ZLI-9019) and hematoxylin counterstaining, while IF staining was performed by Tyramide signal amplification (TSA) method with DAPI reverse staining and observed by confocal microscope (Zeiss, LSM 880).

### Immunohistochemical Quantification

For quantification analyses of PRMT5 protein in human tissue microarray, the integrated optical density (IOD) and area of immunofluorescence were measured by Image-Pro Plus 6.0 software, and the mean optical density (MD) was calculated. In addition, the average number of TFF2-positive cells and GFP-positive cells in single gland were counted from three sections per mouse and repeated in 4 independent mice.

### Real-time quantitative PCR assay

Trizol Reagent (Invitrogen, 15596026) was used to extract total RNAs. 2μg total RNAs were reversely transcribed into cDNAs using a reverse transcription kit (TOYOBO, FSQ-201). Then the cDNAs could then be used as a template for RT-qPCR utilizing the SYBR-Green Master PCR Mix (TOYOBO, QPK-201) and unique primers on a 7500 Fast Real-Time PCR System (Applied Biosystems). Hprt served as the internal control. Table 1 contains the primers ultilized.

### Western blot analysis

RIPA lysis buffer (Applygen, C1053) with phosphatase inhibitors (Roche, 04693159001) and complete mini protease inhibitors (Roche, 04906837001) were used to lyse mouse gastric tissues. Protein concentrations were measured with the Pierce BCA Protein Assay Reagent (Thermo Fisher, 23225). Immunoblotting by Western blot was performed according to standard procedures, using the following antibodies at a concentration of 1:1000. PRMT5 (Abcam, ab109451), H4R3me2s (Abcam, ab5823), CD44 (1:4000, Abcam, ab189524), Cyclin D1 (1:500, Abcam, ab134175), GSK3β (Cell Signaling Technology, 9315), SOSTDC1 (1:100, Abcam, ab99340). Beta Actin (Abcam, ab8227) was served as the internal control. The immunoblot signal was detected and imaged using Enlight Western blotting detection reagents (29100, Engreen Biosystem) and Image Quant LAS 4000 mini (GE healthcare).

### Public databases and survival analysis of human gastric cancer

The TCGA gastric cancer data were downloaded from the cBioPortal, including the DNA alterations and mRNA expression (http://www.cbio portal.org) 22, 23. Kaplan-Meier survival analysis of gastric cancer patients was performed on public microarray data using the Kaplan-Meier Plotter web resource (https://kmplot.com/) 75, 76.

### Statistical analysis

The unpaired, two-tailed Student's t-test was used to evaluate difference between two groups. Survival curves were plotted by Kaplan-Meier estimates and compared by log-rank test. The relative protein level of PRMT5 in human tissue microarray was analyzed by one-way ANOVA test. GraphPad Prism software 8 was used for statistical analysis and data visualization. Data were represented as mean ± SEM. * P < 0.05, ** *P* < 0.01 and *** *P* < 0.001 were used to determine statistical significance for all results.

## Supplementary Material

Supplementary figure.

## Figures and Tables

**Table 1:** Primers used for RT-qPCR analysis

Gene	Primers (5'-3')
Hprt-F	GCTGGTGAAAAGGACCTCT
Hprt-R	CACAGGACTAGAACACCTGC
Prmt5-F	AGCCCATCAAAGCAGCCATT
Prmt5-R	CATTGGGTGGAGGGCGATTT
Lgr5-F	CTTCACTCGGTGCAGTGCT
Lgr5-R	CAGCCAGCTACCAAATAGGTG
c-Myc-F	TCGGGCTCATCTCCATC
c-Myc-R	CACTTGCGGTTGTTGCT
Cyclin D1-F	GAACTACCTGGACCGCTTCC
Cyclin D1-R	CTCCTTCATCTTAGAGGCCACG
CD44-F	GATTCATCCCAACGCTAT
CD44-R	TACTCGCCCTTCTTGCT
Gsk3β-F	CTCATTTCGGCAGACAA
Gsk3β-R	CTCCTTTACCCTCATTACC
Sostdc1-F	AGGGGGAAAGAATTAGCGGC
Sostdc1-R	CCCACTTGAACTCGACTGTTTC

## Abbreviations

PRMT: protein arginine methyltransferase
GSK3: glycogen synthase kinase 3
APC: adenomatous polyposis coli
UEA: ulex europaeus agglutinin
TFF2: trefoil family factor 2
SOSTDC1: sclerostin domain containing 1
RT-qPCR: real-time quantitative PCR
IHC: immunohistochemical
IF: immunofluorescence
