# Supplementary material for: PRMT5 acts as a tumor suppressor by inhibiting Wnt/β-catenin signaling in murine gastric tumorigenesis
Source: Int J Biol Sci. 2022 Jul 4;18(11):4329–40. doi: 10.7150/ijbs.71581 (PMC9295066; doi:10.7150/ijbs.71581)
Supplement: Supplementary file 1 — Supplementary figure. [file ijbsv18p4329s1.pdf]

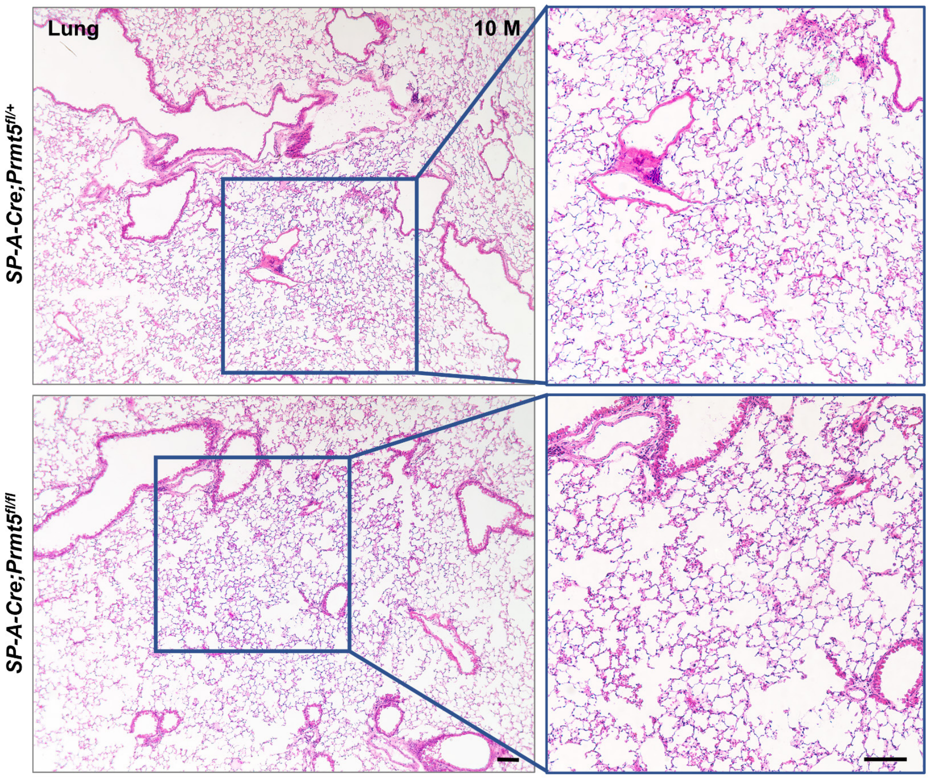

Fig S1. ***Prmt5*** **deletion does not cause any morphological abnormity of mice lung.** Representative H&E staining lung tissue in *Prmt5* mutant mice and control mice at 10 months of age. The blue solid-line boxes were enlarged on the right. Scale bar, 100 μm.
